# Two-photon in vivo imaging reveals cell type-specific mitophagy dynamic changes in mouse somatosensory cortex during aging

**DOI:** 10.1038/s41514-026-00414-5

**Published:** 2026-05-26

**Authors:** Beatriz Escobar-Doncel, Xiaoyi Zhang, Åsne Ljøstad, Mario Fernández de la Puebla, Shreyas Balachandra Rao, Synnøve Algrøy Fjeldstad, Maja Tvedt Dahle, Mahmood Amiry-Moghaddam, Magnar Bjørås, Evandro Fei Fang, Wannan Tang

**Affiliations:** 1 Department of Clinical Molecular Biology, University of Oslo and Akershus University Hospital, Lørenskog, Norway; 2 Department of Molecular Medicine, Institute of Basic Medical Sciences, University of Oslo, Oslo, Norway; 3 Department of Clinical and Molecular Medicine, Norwegian University of Science and Technology (NTNU), Trondheim, Norway; 4 Department of Microbiology, Oslo University Hospital and University of Oslo, Oslo, Norway; 5 The Norwegian Centre on Healthy Ageing (NO-Age) and the Norwegian National Anti-Alzheimer’s Disease (NO-AD) Networks, Oslo, Norway

**Keywords:** Cell biology, Neuroscience, Physiology

## Abstract

Mitochondrial homeostasis is majorly maintained through mitochondrial autophagy (mitophagy). Recent research highlights the region- and cell type-specific nature of mitophagy during brain aging; however, these dynamics have largely remained unexplored in living brains. To address this gap, we conducted two-photon mt-Keima imaging in somatosensory cortical neurons and astrocytes in behaving male mice across two age groups, including 2–3-month-old (early-aged) and 18–20-month-old (old-aged) mice. We show reduced mitophagy in both cell types during aging, and we consistently found a higher level of mitophagy in astrocytes compared to neurons at the same age, in both age groups. Pharmacological augmentation of NAD^+^, a pivotal metabolite that induces mitophagy but normally declines in the aging brain, increased cellular mitophagy in both neurons and astrocytes in old-aged male mice at the dose and method of administration tested. Collectively, our data support an age-dependent reduction of mitophagy in neurons and astrocytes, at least in mouse somatosensory cortex, while NAD^+^ repletion offsets such reduction.

## Introduction

Considering the brain as a highly energy-demanding organ, growing research links dysfunctional mitochondria to the physical changes and neuronal degeneration observed in brain during aging^1–3^. Mitochondria are essential organelles for maintaining cellular energy homeostasis, as well as other fundamental processes such as phospholipid biosynthesis/transport, calcium signaling, ion homeostasis, and even the arbitration of cell death or survival^4–7^. In various model organisms, a reduction in mitochondrial function may contribute to the age-related decline in organ performance, which is a key factor in common neurodegenerative conditions like Alzheimer’s disease^5,8–11^. Consequently, quality control mechanisms have evolved to restore and preserve energy metabolism, especially within the sensitive environment of the brain^10,11^. During the process of selective removal of mitochondria by autophagy (mitophagy), a damaged or superfluous mitochondrion is engulfed by a double-membraned phagophore, which then elongates, matures, and finally fuses with a lysosome for degradation of the engulfed cargo^12^. The molecular mechanisms of mitophagy have also been elucidated in considerable detail in recent years^13–15^.

Various tools exist for measuring mitophagy, including electron microscopy^16^, MitoTimer^17^, co-localization of mitochondrial proteins and autophagic machinery^18^, isolation of autophagic vesicles^19,20^, and ratiometric fluorescent-based probes, such as mito-Rosella^21^, mitoQC^22^, and mt-Keima^23,24^. While these methods have distinct strengths and limitations, ratiometric probes are widely used for ex vivo assessments of mitophagic flux due to their high sensitivity and specificity. However, most measurements using these probes have been performed in fixed tissues^21–23,25^. This approach sacrifices physiological context, as key regulatory signals (e.g., metabolism, temperature) are lost upon tissue extraction, preventing accurate capture of dynamic status of mitophagy regulation and compromising the physiological relevance of the findings^26^. Furthermore, most of the ex vivo brain mitophagy measurements were either cell-type unspecific or targeting only neurons, leaving other cell types in the brain unexplored. Astrocytes, the brain’s major glial cells, perform vital functions such as ion/energy homeostasis, neural plasticity regulation, and blood flow control^27,28^. They play a crucial role in maintaining brain homeostasis, and accumulating evidence also links astrocytes to aging and age-related neurodegenerative diseases, including their metabolic dysfunction and dysregulation of mitophagy^29,30^. Despite existing measurements of neuronal mitophagy ex vivo, astrocytic mitophagy remains largely overlooked.

To address these knowledge gaps and technical challenges, we utilized two-photon imaging with mt-Keima probe in awake, behaving male mice to uncover real-time mitophagy dynamics in layer 2/3 somatosensory cortex (SC). We quantified and compared mitophagy events in both neurons and astrocytes from early-aged and old-aged mice. We also used transmitted electron microscopy (TEM) to investigate the neuronal and astrocytic mitochondrial ultrastructure and identify mitophagy-like events. Finally, we assessed the effect of mitophagy induction through nicotinamide riboside (NR) administration in old-aged mice, revealing cell type-specific responses to therapeutic intervention.

## Results

### Calibration of two-photon laser excitation conditions for in vivo mt-Keima imaging

To explore the real-time mitophagy in the mouse brain, the mt-Keima probe, which is a mitochondria-targeted mKeima^23,31^, was used to illustrate mitophagy events in combination with two-photon imaging in awake behaving mice. The mKeima is a large Stokes shift fluorescent protein with a bimodal excitation spectrum: it peaks at a single-photon excitation wavelength of 440 nm in neutral pH environments (about pH 7) and shifts to approximately 590 nm under acidic conditions (about pH 4), with emission consistently peaking at 620 nm^32,33^. This property allows for dual-excitation ratio imaging to detect autolysosome and mitolysosome formation and their related autophagy or mitophagy events, respectively^23,31^. With two-photon excitation, previous studies have shown that under neutral pH condition the peak excitation wavelength of mKeima is at around wavelength of 880 - 900 nm, and the two-photon absorption (TPA) drops significantly below 800 nm and above 1000 nm^34,35^, however, no similar data was available under acid condition.

**Fig. 1:**
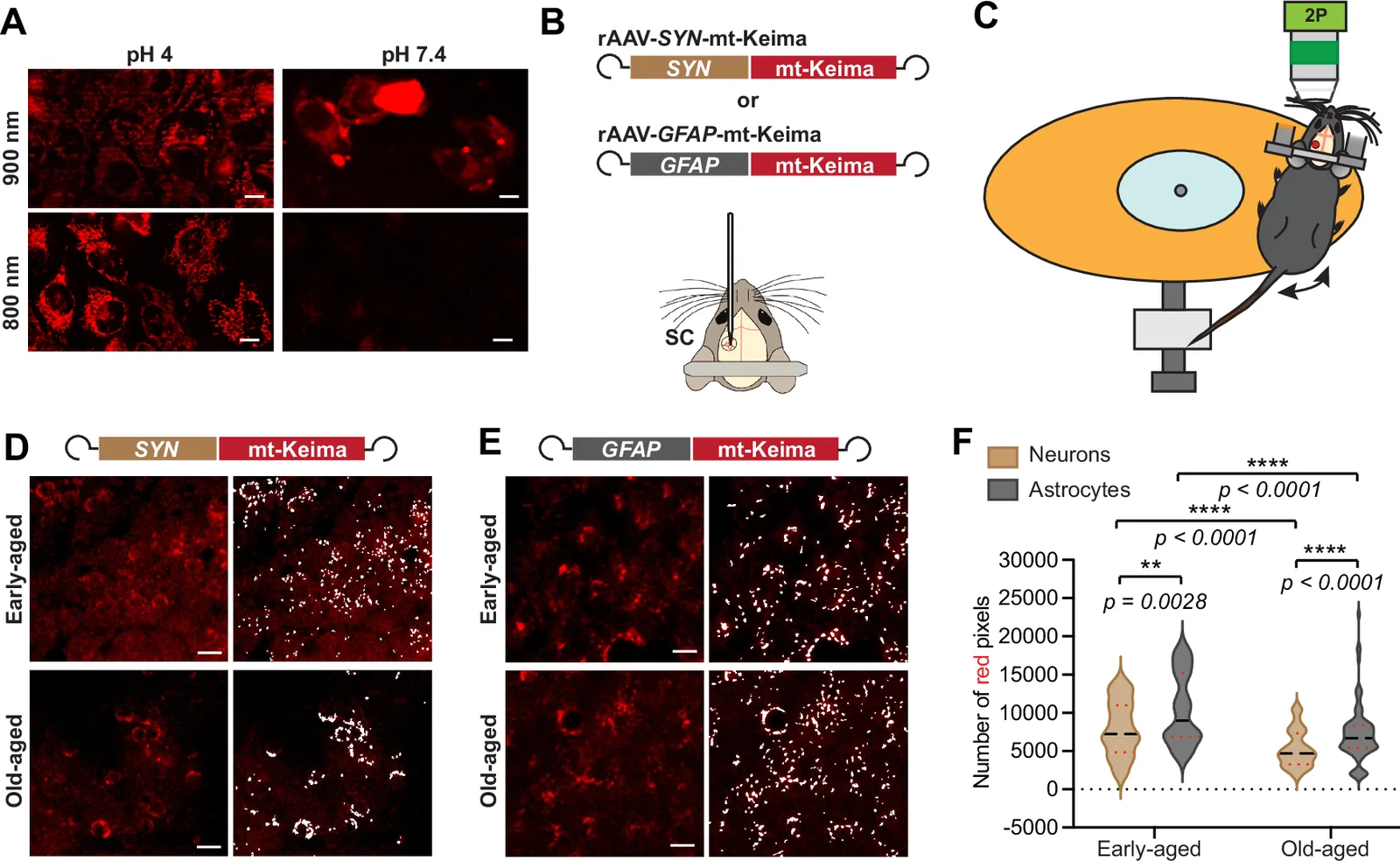
Two-photon mt-Keima imaging in neurons and astrocytes during aging. **A** Two-photon imaging of mt-Keima expressing Hela cell line under acidic (pH 4) and neutral (pH 7.4) environment with two-photon excitation wavelengths at 900 nm and 800 nm. Scale bar, 10 μm. **B** Illustration of rAAV constructs expressing mt-Keima under *SYN* and *GFAP* promoters, and schematic representation of rAAV delivery in mouse somatosensory cortex (SC). **C** Illustration of the two-photon (2 P) imaging setup in head-fixed awake behaving mice. Representative images (left side images in each group) of rAAV-*SYN*-mt-Keima (**D**) and rAAV-*GFAP*-mt-Keima (**E**) transduced SC in early-aged and old-aged wild-type mice. Signal extraction for quantitative analysis using *Ilastik* software is shown on the right in each group. Scale bar, 10 µm. **F** Quantitative comparison of detected mt-Keima signals indicating mitophagy levels in neurons and astrocytes during aging. Early-aged group, rAAV-*SYN*-mt-Keima, *n* = 60 from 3 mice; rAAV-*GFAP*-mt-Keima, *n* = 80 from 6 mice; old-aged group, rAAV-*SYN*-mt-Keima, *n* = 69 from 5 mice; rAAV-*GFAP*-mt-Keima, *n* = 94 from 3 mice. Statistical significance was tested by the Mann-Whitney test. Medians and quartiles are indicated by black and red dotted lines, respectively; ns, non-significant; *p* < 0.05 (*); *p* < 0.01 (**); *p* < 0.001 (***); *p* < 0.0001 (****).

Since sequential two-photon scanning with two distinct excitation wavelengths is technically challenging for dynamic imaging in live, behaving mice, we sought the most suitable single excitation two-photon wavelength that maximizes mt-Keima signals from acidic environments (functional mitolysosomes) while minimizing mt-Keima signals from neutral environments. To achieve this, we used a HeLa cell line stably expressing mt-Keima^23^. Given the limited laser power output and low TPA above 1000 nm, we tested wavelengths between 800 nm and 900 nm. Using 800 nm excitation wavelength, we achieved maximum mt-Keima fluorescent signals at pH 4 and minimal signals at pH 7 in these Hela cells (Fig. 1A**)**. This wavelength effectively ensured that the majority of the captured signal originated from the acidic environment of active mitolysosomes. Thus, the 800 nm two-photon excitation wavelength was selected to perform the following in vivo imaging experiments.

### Neurons and astrocytes exhibited distinct mitophagy dynamics during aging

To measure mitophagy levels in both neurons and astrocytes, *SYN* and *GFAP* promoters were used to drive expression of mt-Keima in neurons and astrocytes via recombinant adeno-associated virus (rAAV) gene delivery, respectively (Fig. 1B), and both promoters show cell type-specificity of neurons and astrocytes in mouse SC region (Figure S1A). To reveal mitophagy levels in neurons and astrocytes during aging, two weeks after rAAV transduction, both early-aged (2–3-month-old) and old-aged (18–20-month-old) male adult wild-type mice expressing mt-Keima in either neurons or astrocytes in layer 2/3 SC were placed on a freely spinning disc for imaging (Fig. 1C–E, Video S1–S4). We quantified mitophagy levels based on the absolute mt-Keima signals acquired from layer 2/3 SC neurons or astrocytes within defined regions of interest (ROIs) of fixed dimensions (Fig. 1D, E, right panels). Our data revealed two key findings regarding mitophagy dynamics (Fig. 1F). Firstly, we observed that mitophagy levels decreased significantly with age in both cell types. Old-aged mice exhibited approximately a 34% reduction in neuronal mitophagy and a 31% reduction in astrocytic mitophagy compared to the early-aged group. Secondly, in both age groups, astrocytes consistently maintained significantly higher mitophagy levels than neurons (approximately 31% higher in early-aged and 36% higher in old-aged mice). Collectively, our in vivo characterization of neuronal and astrocytic mitophagy in the live mouse SC suggests that these cell types follow two distinct dynamic patterns during the aging process.

### Aging affected mitochondrial features in neurons and astrocytes

**Fig. 2:**
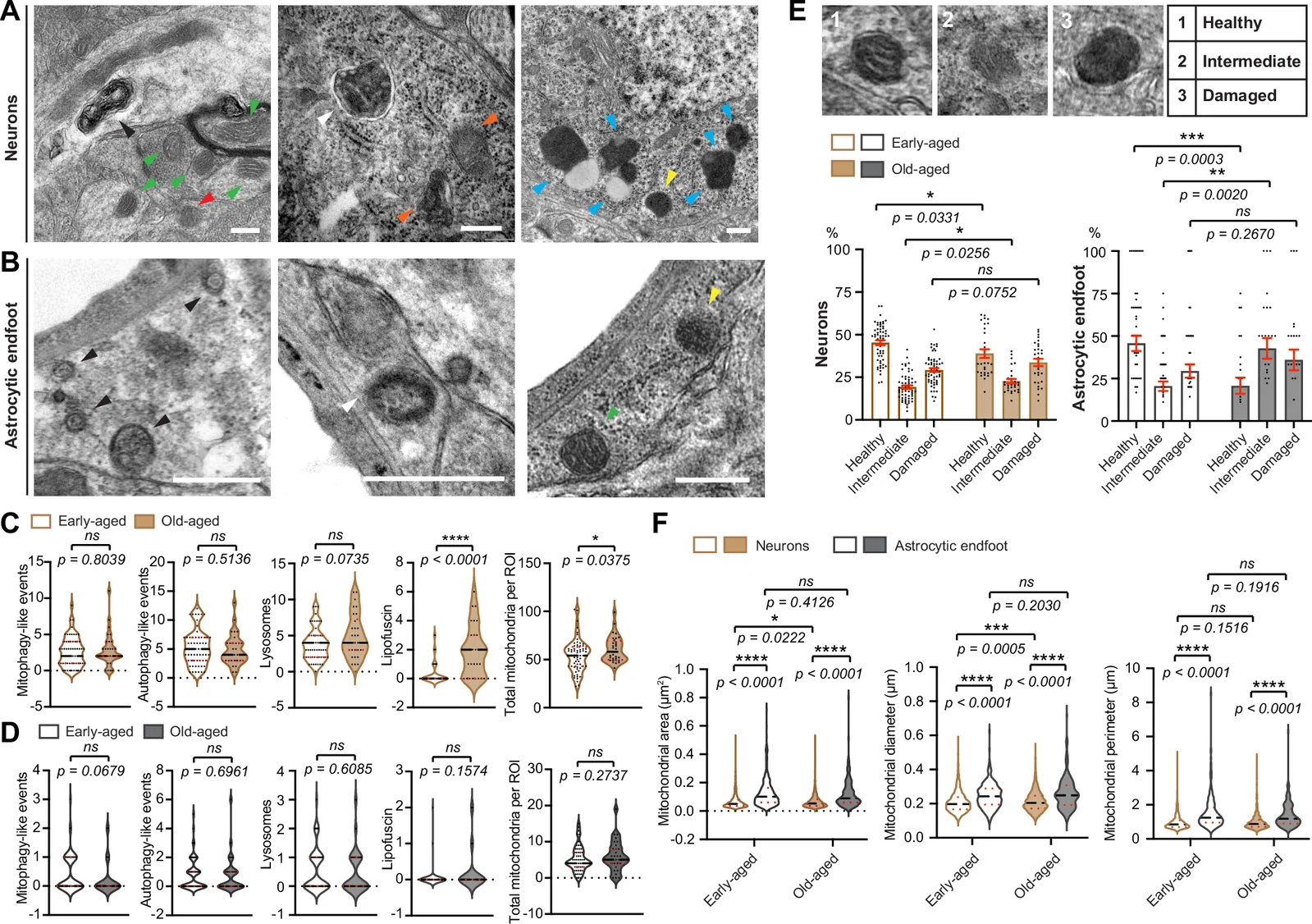
TEM analyses on mitochondrial features during aging. Representative TEM images of neurons (**A**) and astrocytic endfeet abutting the blood vessels (**B**). Green, orange and red arrowheads indicate “healthy”, “intermediate” and “damaged” mitochondria, respectively; black, white, yellow and blue arrowheads indicate mitophagy-like events, autophagy-like events, lysosomes and lipofuscin, respectively. Scale bars, 500 nm. Comparison of mitophagy-like events, autophagy-like events, lysosomes, lipofuscin and total mitochondria per ROI (Welch’s *t*-test) in neurons (**C**) and astrocytic endfeet (**D**) during aging. Neurons, early-aged, *n* = 63 from 6 mice, old-aged, *n* = 32 from 3 mice; astrocytes, early-aged, *n* = 60 from 6 mice, old-aged, *n* = 31 from 3 mice. (**E**) Classification and comparison of mitochondrial status in neurons and astrocytic endfeet during aging. Neuronal “damaged” group, astrocytic endfoot “healthy” and “intermediate” groups, Welch’s t-test. Neurons, early-aged, *n* = 63 from 6 mice; old-aged, *n* = 32 from 3 mice; astrocytic endfeet, early-aged, *n* = 57 from 6 mice, old-aged, *n* = 28 from 3 mice. (**F**) Comparison of mitochondrial areas, diameter and perimeter during aging in neurons and astrocytes. Neurons, early-aged, *n* = 2971 from 6 mice; old-aged, *n* = 1710 from 3 mice; astrocytic endfeet, early-aged, *n* = 307 from 6 mice; old-aged, *n* = 182 from 3 mice. Statistical significance was tested by Mann-Whitney test, unless stated otherwise. Medians and quartiles are indicated by black and red dotted lines, respectively; ns, non-significant; *p* < 0.05 (*); *p* < 0.01 (**); *p* < 0.001 (***); *p* < 0.0001 (****).

To complement our two-photon imaging data, we used traditional TEM to assess mitochondrial ultrastructure in the SC regions of the same early- and old-aged mice (Fig. 2A, 2B). We quantified mitophagy/autophagy-like events, total numbers of lysosomes, lipofuscin and mitochondria per ROI (Figs. 2C and 2D). Furthermore, we assessed morphological integrity of all identified mitochondria across cell types. Mitochondria were classified into three categories: “healthy” (intact, sharply defined cristae), “intermediate” (partially degenerated cristae), and “damaged” (lack of well-defined cristae) (Fig. 2E). Finally, we measured specific mitochondrial morphological parameters—including size, diameter, and perimeter—in both neurons and astrocytes (Fig. 2F). To ensure cell-type specificity in TEM, astrocytic quantification was limited strictly to endfeet in direct contact with blood vessels (Fig. 2B).

We observed no significant differences in mitophagy/autophagy-like events or total lysosome counts within either neurons or astrocytic endfeet. However, the accumulation of lipofuscin was significantly higher in the neurons of aged mice, indicating characteristic age-related morphological changes in the brain (Fig. 2C, 2D). During aging, the total number of mitochondria increased in neurons (Fig. 2C), but remained unchanged in astrocytic endfeet (Fig. 2D). In both cell types, the proportion of “healthy” mitochondria was significantly higher in the early-aged group. In contrast, the old-aged mice showed a significant shift towards “intermediate” mitochondrial states, while the proportion of “damaged” mitochondria remained constant across both age groups (Fig. 2E). The measurement of mitochondrial parameters showed that the neuronal mitochondrial areas and diameters became significantly larger (Fig. 2F), suggesting age-dependent morphological changes in neurons. In astrocytic endfeet, the total number of mitochondria and morphological details remained similar during (Fig. 2E, 2F). Regardless of age groups, the astrocytic mitochondrial areas, diameters and perimeters were all significantly larger than those in neurons (Fig. 2F and Table 1), which may reflect an age-independent, cell-type-specific mitochondrial morphological characteristics in mouse SC.

**Table 1:** Mitochondrial parameter comparison in neurons and astrocytes with TEM

	Mitochondrial parameter	Neurons (mean ± SEM)	Astrocytes (mean ± SEM)	*p*-value (Mann-Whitney test)
**Early-aged group**	Area	0.068 ± 0.001	0.128 ± 0.006	*p* < 0.001
	Diameter	0.209 ± 0.001	0.245 ± 0.004	*p* < 0.001
	Perimeter	0.986 ± 0.009	1.506 ± 0.052	*p* < 0.001
**Old-aged group**	Area	0.069 ± 0.001	0.122 ± 0.007	*p* < 0.001
	Diameter	0.214 ± 0.001	0.261 ± 0.007	*p* < 0.001
	Perimeter	0.994 ± 0.011	1.380 ± 0.053	*p* < 0.001

### NR treatment improved neuronal and astrocytic mitophagy in old-aged mice

**Fig. 3:**
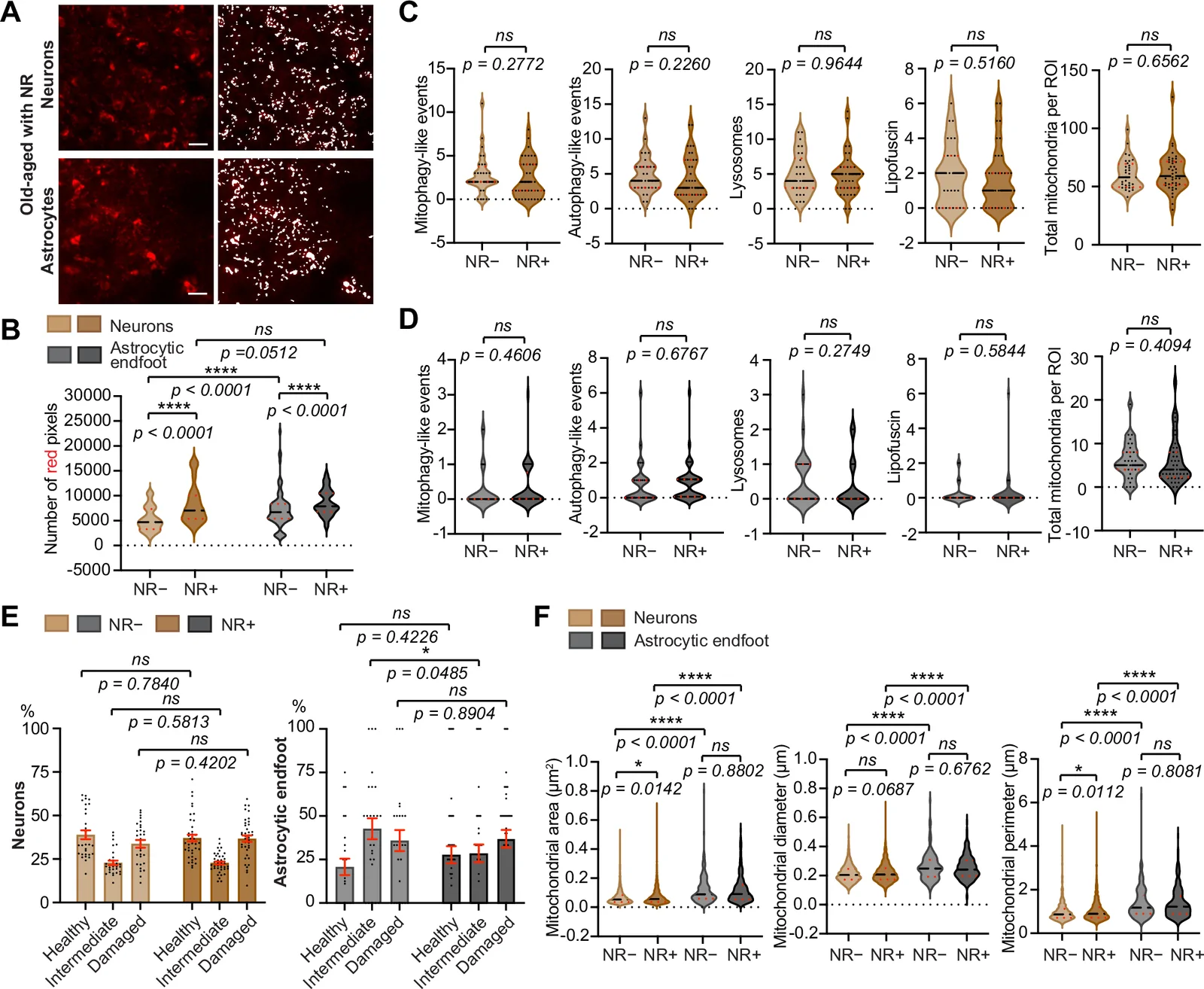
Nicotinamide riboside (NR) treatment effects on neurons and astrocytes in old-aged mice. **A** Representative images of rAAV-*SYN*-mt-Keima and rAAV-*GFAP*-mt-Keima transduced neurons and astrocytes, respectively, in SC in old-aged adult mice with NR treatment (left images). Signal extraction for quantitative analysis using *Ilastik* software were showing on the right. Scale bar, 10 µm. **B** Quantitative comparison of detected mt-Keima signals indicating mitophagy levels in neurons and astrocytes before and after NR treatment. Group without NR, rAAV-*SYN*-mt-Keima, *n* = 69 from 5 mice; rAAV-*GFAP*-mt-Keima, *n* = 94 from 3 mice; group with NR, rAAV-*SYN*-mt-Keima, *n* = 65 from 5 mice; rAAV-*GFAP*-mt-Keima, *n* = 90 from 3 mice. Comparison of mitophagy-like events, autophagy-like events, lysosomes, lipofuscin and total mitochondria per ROI in neurons (**C**) and astrocytic endfeet (**D**) before and after NR treatment. Neurons, group without NR, *n* = 32 from 3 mice, group with NR, *n* = 41 from 4 mice; astrocytic endfeet, group without NR, *n* = 31 from 3 mice, group with NR, *n* = 40 from 4 mice. **E** Comparison of mitochondrial status in neurons and astrocytic endfeet before and after NR treatment. Astrocytic endfoot “damaged” group, Welch’s t-test. Neurons, group without NR, *n* = 32 from 3 mice, group with NR, *n* = 41 from 4 mice; astrocytic endfeet, group without NR, *n* = 28 from 3 mice, group with NR, *n* = 38 from 4 mice. (**F**) Comparison of mitochondrial areas, diameter and perimeter in neurons and astrocytes before and after NR treatment. Neurons, group without NR, *n* = 1710 from 3 mice; group with NR, *n* = 2295 from 4 mice; astrocytic endfeet, group without NR, *n* = 182 from 3 mice; group with NR, *n* = 231 from 4 mice. Statistical significance was tested by Mann-Whitney test, unless stated otherwise. Medians and quartiles are indicated by black and red dotted lines, respectively; ns, non-significant; *p* < 0.05 (*); *p* < 0.01 (**); *p* < 0.001 (***); *p* < 0.0001 (****).

To further apply the two-photon mt-Keima in vivo imaging to a therapeutic context, we investigated a pharmacological activation of mitophagy using NR. NR is known to enhance mitophagy by increasing nicotinamide adenine dinucleotide (oxidized form, NAD^+^) levels, which in turn activates NAD⁺-dependent pathways that promote autophagy and mitophagy. This activation stimulates both the post-translational regulation of related proteins and the transcriptional regulation of genes encoding proteins involved in these pathways^8,36–38^. We aimed to determine whether NR could ameliorate the age-dependent decline of brain mitophagy in vivo. Old-aged male mice previously imaged were treated with 12 mM NR for 3 weeks and subsequently re-imaged using two-photon microscopy (Fig. 3A). The NR treatment significantly increased mitophagy levels in both neurons (56% improvement) and astrocytes (19% improvement), indicating an enhancement in mitochondrial removal mechanisms (Fig. 3B). However, subsequent TEM analysis of the SC tissues showed that the NR treatment did not significantly alter mitophagy-like/autophagy-like events, total numbers of lysosomes, lipofuscin and mitochondria per ROI (Fig. 3C, 3D). Across both cell types, the distribution of mitochondrial classes remained largely consistent following NR treatment; however, the proportion of “intermediate” mitochondria significantly decreased specifically within astrocytic endfeet in the NR-treated group (Fig. 3E). In addition, neuronal mitochondrial sizes measured by area and perimeter were significantly increased after the NR treatment, whereas those in astrocytic endfeet did not (Fig. 3F), suggesting changes in neuronal mitochondrial morphological characteristics following NR intervention.

## Discussion

Aging is characterized by a progressive decline in cellular function, with both mitochondrial dysfunction and impaired autophagy emerging as central hallmarks in research concerning brain aging and age-related neurodegenerative diseases^1,39,40^. In this study, we present the first real-time in vivo two-photon imaging assessment of cell type-specific mitophagy in the SC of early-aged and old-aged male adult mice. Our findings revealed distinct patterns of mitophagy dynamics in neurons and astrocytes during the aging process. Specifically, we observed that mitophagy levels, as measured by mt-Keima reporter in the SC, were significantly reduced with age in both cell types. Crucially, astrocytic mitophagy levels were consistently higher than neuronal levels in both age groups (Fig. 1F). These data underscore the need for more comprehensive approaches to study brain metabolism during aging that account for distinct contributions of both neuronal and glial populations.

This study offered a significant technical advantage, providing higher accuracy in describing the real-time dynamics of mitophagy in the brain compared to previously published research. The employment of two-photon microscopy has fundamentally improved in vivo experimental conditions of using mitophagy reporters. When compared to imaging of mt-Keima in acute brain slices^23^ or confocal imaging of mt-QC in the fixed transgenic mouse brain^25,41^, our approach restored the real-time mitophagy dynamics in living mouse brains. To our surprise, we did not observe fast temporal dynamics of mitophagy during imaging. Instead, the recorded mitophagy pattern remained relatively stable in both neurons and astrocytes over five-minute recording period (Video S1 - S4).

In this study, the rAAV delivery of the mitophagy reporter, the mt-Keima probe, has achieved cell type-specific expression that has not been very clearly illustrated in previous studies using transgenic mice. Transgenic mt-QC mice typically use a CAG promoter, which primarily drives expression in neurons^22,25,41^, but also may result in expression in astrocytes and oligodendrocytes^42^. While Schmid et al. demonstrated neuronal-specific expression of mt-QC in *Drosophila*^43^, their analysis was performed on fixed brain tissue. Direct comparison between the results from two species and in vivo versus ex vivo experimental conditions is challenging. Thus, our results on measuring mitophagy levels using two-photon microscopy in the SC provide a valuable perspective on cell type specificity of brain mitophagy.

While the use of mt-Keima allows for real-time in vivo mitophagy assessment, it does not provide information on status of healthy and impaired mitochondria. Our TEM analyses, offering subcellular resolution, helped to clarify this property at the 2D ultrastructural level, although a more comprehensive assessment of mitochondrial morphology would require electron tomography or serial sectioning. The TEM results showed that the total count of neuronal mitochondria was increased during aging, and the number of *healthy* mitochondria was decreased in both neurons and astrocytic endfeet (Fig. 2C, 2E, respectively). As previously reported in the *Drosophila* brain^43^, this also suggests that mitochondrial quality control is a more critical determinant of brain aging than the simple loss of mitochondrial content. Meanwhile, we also observed that the actual size of mitochondria in astrocytic endfeet was larger than in neurons (Fig. 2F). Functionally, astrocytes possess several microcompartments with distinct activation responses, as measured by changes in intracellular calcium concentration^44,45^. Astrocytic endfeet are one distinct functional microcompartments that participate in forming the blood-brain barrier, and their specific metabolic demands might differ from mitochondria in other microcompartments of astrocytes. Although we should not generalize our findings in the defined endfoot compartment to the entire astrocyte, these data, combined with the in vivo two-photon mt-Keima imaging, suggest that the distribution of mitochondria and metabolic demands likely differ between neurons and astrocytes, opening new avenues to understand the contribution of spatial and temporal changes in mitochondria during aging.

Although our TEM analyses did not fully recapitulate the two-photon imaging results, this discrepancy likely results from both technical and biological differences between the two readouts. Two-photon imaging with mt-Keima specifically reports mitochondria that have reached an acidified compartment (mitolysosomes), effectively bypassing earlier stages of the pathway. Conversely, TEM identifies mitophagic structures based solely on 2D morphology; however, because mitochondrial remnants in late-stage autolysosomes are often indistinguishable from other cargo, TEM likely underestimates the total number of mitophagic events. Consequently, these two methods provide distinct but complementary snapshots of the mitophagic process. In addition, when compared to the areas covered by two-photon imaging, the TEM sample sizes were small (3–6 mice per group, approximately 10 images per cell type per mouse), which limited the statistical power. This limitation was particularly evident for the analysis intended for astrocytes, as we were restricted to performing TEM analysis only on astrocytic endfeet to ensure cell type-specificity. Expanding the TEM study cohort and incorporating functional assays of mitochondrial activity would strengthen these conclusions.

Furthermore, we have demonstrated that pharmacological activation of brain mitophagy with NR elevates mitophagy levels in both neurons and astrocytes in layer 2/3 in the mouse SC. The beneficial effects of NAD^+^ repletion in neurons have previously been shown in aging and disease models, resulting in notable increases in mitophagy and mitochondrial function^38,46–49^. Importantly, NR treatment has been shown to increase NAD^+^/NADH ratio in the brain^47^. While it is hard to predict the precise mechanistic basis for these observed effects of NR treatment, potential factors may involve NAD^+^ metabolism, lysosomal function, or mitophagy regulatory pathways in neurons and astrocytes^50,51^. The mechanistic effects of NR have been explored in multiple cell types^52^. Previous studies have linked the NAD^+^-dependent SIRT-1 as a mediator of the NR effects in neurons, but much less is known on astrocytes^8,53^. Follow-up mechanistic and SC-dependent behavioral studies are urgently needed to fully understand treatment effect and functional significance of elevating mitophagy levels via this intervention.

Due to the technical limitations of two-photon microscopy, traditional ratiometric imaging requiring two distinct excitation wavelengths was not feasible. Instead, we used an optimal single two-photon excitation wavelength of 800 nm, which maximized mt-Keima signals in acid environment while minimizing signals at neutral pH (Fig. 1A). Given that the mt-Keima signals detected at pH 7.4 were very low, and recordings were performed under similar conditions, we considered 800 nm excitation suitable for measuring in vivo mt-Keima signals under acid conditions. Under standardized recording conditions in awake mice, maintaining consistent laser power and photomultiplier tube (PMT) gain, the mt-Keima signal in the red channel is substantially more robust than the signal in the green channel at 800 nm. This in vivo observation is consistent with our findings in the mt-Keima HeLa cell line (Fig. 1A), confirming that the excitation efficiency at this specific wavelength heavily favors the red emission, thereby tolerating the remaining minimum green signals. Interestingly, we observed a previous unreported change in the mt-Keima emission spectrum when exciting with 800 nm versus 900 nm wavelengths. With higher power output at 800 nm compared to 900 nm from the two-photon laser, signals collected in the green emission channel at pH 4 and pH 7.4 were stronger at 900 nm excitation (Figure S1B). This indicates an emission spectrum shift when changing excitation wavelength, which has not been reported from previous studies with mt-Keima using both single photon and two-photon excitation^32–35^. Due to these minimum green emitted signals, we could not use the green channel to normalize the mt-Keima signals. To mitigate potential variability in cell density across different imaging planes and given that total cell counts in this region remain stable during aging in male mice^54–57^, we quantified puncta per defined ROI and analyzed multiple fields of view across diverse areas of layer 2/3 of the SC. Nonetheless, cortical thinning and/or cell loss have been reported during aging in layer 5/6 of the sensory cortex, though notably not in layers 2/3^58^, we cannot completely exclude the possibility that subtle cortical thinning and associated cell loss could influence our measurements in the SC, and thus, represent a potential confounding factor that was not directly corrected for in our analysis due to technical constraints. A further technical limitation is that when imaging the miniature structure of mitochondria, the spatial resolution obtained from two-photon microscopy was relatively poor when compared to previous studies using stimulated emission depletion microscopy^23^, and individual mitochondria could not be illustrated. Additionally, the limited imaging depth of our method restricted our access to neurons and astrocytes in layer 2/3 of the SC. Therefore, the applicability of our conclusions to other brain regions, particularly the subcortical areas, required further investigation.

Several key questions remain unanswered and require future investigation. First, our data reported were all from male mice, and it is crucial to assess whether gender differences exist in mitophagy regulation and in response to NR treatment. Notably, previous research has reported sex-dependent mitophagy differences in cerebellar astrocytes, with higher mitophagy in females during midlife^25^; similarly, there were sex- and region-specific shifts in chaperone-mediated autophagy with aging^59^. Second, while we explored changes in mitophagy in neurons and astrocytes during aging in live mice, we did not include microglia. Third, building on the success of this study, in vivo two-photon microscopy-based mitophagy studies should be extended to other brain regions of live mice. Fourth, because our in vivo imaging setup does not allow quantification of the total mitochondria content per cell type, our measurements reflect absolute mitophagy events rather than relative mitophagy. As a result, potential age- or cell type-dependent differences in mitochondria could influence our readouts and should be addressed in the future. Fifth, while our study focused primarily on structural and morphological changes, the functional and behavioral implications of the age-related astrocytic and neuronal mitophagy decline remain unclear. Future work should examine whether age-related astrocytic mitophagy deficits directly contribute to any neuronal dysfunction, synaptic loss, or even cognitive decline. In addition, growing evidence links mitochondrial status with neuronal activity^60^. Future studies should address whether the decline in mitophagy is a consequence of decreased neuronal activity during aging. Lastly, cell type-specific functional studies on mitochondria in early-aged and old-aged mice, as well as after NR treatment, are still lacking. Direct measurements of mitochondrial function (*e.g*., respiratory capacity, membrane potential, or ATP production) will be crucial to validate whether the structural alterations and changes in mitophagy that we observe reflect mitochondrial damage, and to better understand the potential of NR as a therapeutic agent.

## Methods

### Animals

Male mice with age of 2–3-month-old (early-aged) and 18–20-month-old (old-aged) were housed with a 12-hour light/dark cycle (light on at 8 a.m.) with running wheels and cardboard houses et to enrich the housing environment. All animal handling and experimental procedures were performed according to the European Animal Research law (Directive 2010/63/EU) and approved by the Norwegian Food Safety Authority (project number: FOTS #27090 and #29730).

### Nicotinamide Riboside (NR) treatment

After two-photon imaging, all old-aged mice were administrated with NR (12 mM NR in drinking water) for a duration of 3 weeks. To ensure consistency and maintain NR stability, drinking water was replaced once per week in both groups throughout the treatment period.

### Plasmid construction and virus production

The DNA sequence of the mt-Keima^23^ was synthesized and subcloned into the recombinant adeno-associated virus (rAAV) vector pAAV-6P-SEWB^61^ and pAAV-GFAP-GCaMP5E^45^ with 5’ BamHI and 3’ HindIII to create pAAV-*SYN*-mt-Keima and pAAV- *GFAP*-mt-Keima, respectively. Serotype 2/1 rAAVs from pAAV-*SYN*-mt-Keima and pAAV-*GFAP*-mt-Keima were produced and purified by AVB Sepharose affinity chromatography^62^, following titration with real-time PCR (rAAV titer about 1.0–6.0 × 10^12^ viral genomes/mL, TaqMan Assay, Applied Biosystems).

### Surgical procedures and virus transduction

Mice were briefly anesthetized with 3% isoflurane in an induction chamber and then quickly fastened on a stereotaxic frame with a nose cone (Model 963, KOPF Instruments). Animals were maintained under anesthesia with 1.2–1.5% isoflurane in a mixture of 80% oxygen and 20% room air and buprenorphine 0.1 mg/kg s.c. preemptively for analgesia. Before the surgery, Bupivacaine was administered subcutaneously over the skull and left for 10 min. Skin and connective tissues over the somatosensory cortex were removed. A custom-made titanium head-bar was glued to the mouse skull using cyanoacrylate glue (Loctite Super Glue) and sealed with dental cement (Meliodent, Kulter). A 2.5-mm-diameter craniotomy was then drilled with its center coordinates relative to Bregma of anteroposterior (AP) -1.1 mm and mediolateral (ML) 3.5 mm. A fine dental drill was used to carefully cut the skull with intermittent soaking and removal of debris until only ~0.1 mm of the bone thickness was left. After 10 min of soaking in 0.9% saline, the bone flap was carefully removed. The rAAVs were slowly injected (20–30 nL/min) at three evenly spaced locations (70 nL each at 400 μm depth) positioned to stay clear from large blood vessels. A glass plug consisting of two coverslips with diameters of 2.5 and 3.5 mm glued together with ultraviolet-curing glue (Norland Optical Adhesive No. 61) was placed and fixed on the craniotomy using cyanoacrylate-gel glue (Loctite Super Glue Gel) and reinforced with dental cement (Meliodent, Kulter). The glass window was slightly pressing on the dura to prevent dural overgrowth. Mice with implant complications were excluded from further experiments.

### Two-photon imaging

Three to seven weeks after the surgery, mice were imaged under a two-photon microscope (Ultima IV, Bruker/Prairie Technologies) with a 16 × 0.8 NA water-immersion objective (model CFI75 LWD 16XW, Nikon), an InSight DeepSee laser (Spectra-Physics) and Peltier-cooled photomultiplier tubes (model 7422PA-40, Hamamatsu Photonics K.K). Optical filter settings: a main dichroic filter (ZT473-488/594/NIRtpc) in the detection unit reflects the collected light. The light enters the detector house with four channels, passing a ZET473-488/594/NIRm filter to shield the photo multiplier tubes from the reflective light. Inside the detector house, the light is split into two fractions separated at the wavelength of 560 nm by a dichroic filter (T560lpxr). The green wavelength light is further guided by a secondary dichroic beam splitter at 495 nm (T495lpxr) and filtered by an ET525/50m-2p bandpass filter. And the red wavelength light is similarly guided by a secondary beam splitter at 640 nm (T640lpxr) and subsequently filtered by an ET595/50m-2p bandpass filter. The emitted photons were detected with Peltier-cooled photomultiplier tubes (model 7422PA-40 by Hamamatsu Photonics K.K.). After calibration, the excitation wavelength of 800 nm was used to record mt-Keima signals. Images with 512 × 512 pixels resolution were acquired at about 1 Hz. Imaging time series of 5 min were recorded from multiple fields of view (FOVs) in layer 2/3 of somatosensory cortex of each animal.

### Imaging analysis

Imaging raw data were corrected for motion artifacts using the NoRMCorre movement correction algorithm^63^. Recordings that could not be sufficiently motion-corrected were excluded from the analysis. Subsequently, the stabilized T-series were imported into ImageJ for further processing. From each T-series, sets of five consecutive frames were stacked to generate one average Z-projection image, corresponding to 5 seconds of recording time (1 Hz), with inclusion criteria requiring uninterrupted signal quality (i.e. noise recording) across all five frames. Up to five sets (5-frame stack) were selected per stabilized T-series with 1 min time intervals from the recording, ensuring a complete representation of a full recording. The same sets were selected for both channels to guarantee consistency. Each five-frame stack was then converted into a single image by calculating the average intensity projection. To minimize edge artifacts (e.g. noise recording) and ensure consistent image dimensions across the dataset, all images were uniformly cropped, irrespective of the presence of visible artifacts.

### mt-Keima analysis by Ilastik

All images were further processed using Ilastik, an open-source image classification and segmentation software (www.ilastik.org)^64^. Ilastik applies supervised machine learning algorithms to rapidly quantify image features, enabling the establishment of consistent thresholding parameters across all images. Feature selection included color/intensity (*σ* = 0.3, 0.7, 1.00, 1.60, 3.50, 5.00, and 10.00), edge (*σ* = 0.7, 1.6, and 5.00), and texture (*σ* = 1.00, 3.50, and 10.00). Pixel classification was then performed to distinguish background noise (“background” label) from true signal (‘pixel’ label). Up to five sets of images per condition were used to train the classifier. Thresholding for the ‘pixel’ label was applied using the simple method, with smoothing parameters set to 0.0 for both minimum and maximum values, and a threshold value of 0.45. A size filter was also applied, with the minimum object size set to 9 pixels, corresponding approximately to the size of a mitochondrion. Following training and thresholding, experimental images were batch-processed, and results were manually inspected. After the batch processing, object information was exported for downstream quantification, including total pixel number, object count, and object feature analysis. These outputs were then used for subsequent ratiometric quantification.

### Sample preparation and embedding for TEM

For perfusion and tissue preparation, mice from all groups were deeply anesthetized using 5% isoflurane and then were transcardially perfusion fixed, initially using 2% dextran in 0.1 M phosphate buffer for 20 s followed by the fixative for 20 min. The fixative consisted of 4% formaldehyde and 0.1% glutaraldehyde in 0.1 M phosphate buffer at pH 7.4. Following perfusion, the brains were carefully dissected out post fixed overnight in the fixative. The following day, the brains were stored in a 1:10 dilution of the fixative in 0.1 M phosphate buffer until further use. The samples were embedded for electron microscopy as follows: small blocks of volume ~1 mm^3^ from tissue were dissected out from the fixed brains and were subjected to freeze-substitution procedure as described previously^65^. Briefly, tissue blocks were cryoprotected in 4% glucose for overnight, followed by suspension in graded glycerol solution (10%, 20% and 30% glycerol in 0.1 M phosphate buffer) for 30 min in each gradient, with the final step being overnight suspension in 30% glycerol. The following day, tissues were rapidly frozen in propane cooled to −170 °C using liquid nitrogen before being subjected to freeze substitution. Samples were later embedded in methacrylate resin (Lowicryl HM20) and polymerized by UV irradiation at −45 °C for 48 h. Ultrathin sections of thickness 80–90 nm were cut and placed on formvar carbon-coated support film in Ni-grids and counterstained using 2% uranyl acetate and 0.3% lead citrate for 90 s each. Images were acquired using a Tecnai 12 electron microscope (FEI) at 80 kV.

### TEM analysis and quantification

TEM images of neurons and astrocytic endfeet were analyzed for the quantification of mitochondria number and classification, mitophagy and autophagy events. The region of interest (ROI) for neurons included soma, synapses, dendrites and axons, and for astrocytes included endfeet that abut the blood vessels. The general approach for TEM analysis of the brain was based on previously published references (Introduction » Fine Structure of the Aging Brain | Boston University). Mitochondria were classified based on structural criteria: mitochondria with intact and sharp defined cristae were considered *healthy*; those having partial degenerated cristae were defined as *intermediate*; those displaying no well-defined cristae were categorized as *damaged*; and structures where these features could not be confidently identified were labeled as *unrecognized*. Quantification of mitophagy, autophagy, lysosomes and lipofuscin events were performed according to established protocols^66–68^.

### Statistics

Statistical analysis was performed using Prism (Version 9.3.1 for Mac OSX, GraphPad Software). Details about the choice of statistics are described in the main text and figure legends. Full descriptions of statistical parameters were accessed with the original data before choosing a suitable analysis.

## Supplementary information

Supplementary Information

Video-S1.

Video-S2.

Video-S3.

Video-S4.

## Data Availability

The original recorded data are available upon requests by the lead contact Wannan Tang (wannan.tang@ntnu.no).

## Code availability

All original code for image pre-processing has been deposited at GitHub and is publicly available at 10.1016/j.jneumeth.2017.07.031. The mitophagy quantification was performed using an open-source image classification and segmentation software Ilastik (www.ilastik.org).
